# CRABPII和E-FABP在非小细胞肺癌中的表达及其意义

**DOI:** 10.3779/j.issn.1009-3419.2013.01.03

**Published:** 2013-01-20

**Authors:** 倩 刘, 世凤 王, 缓 徐, 尚福 张

**Affiliations:** 610041 成都，四川大学华西医院病理科 Department of Pathology, West China Hospital, Sichuan University, Chengdu 610041, China

**Keywords:** 肺肿瘤, CRABPII, E-FABP, 预后, 组织芯片, 免疫组化, Lung neoplasms, CRABPII, E-FABP, Prognosis, Tissue microarray, Immunohistochemistry

## Abstract

**背景与目的:**

细胞视黄酸结合蛋白（cellular retinoic acid-binding protein Ⅱ, CRABPⅡ）和表皮型脂肪酸结合蛋白（epidermal fatty acid-binding protein, E-FABP）作为维甲酸（retinoic acid, RA）的转运蛋白，通过RA信号传导通路，从正反两方面影响细胞的增殖和凋亡。本研究旨在探讨CRABPII和E-FABP在非小细胞肺癌（non-small cell lung cancer, NSCLC）中的表达及意义。

**方法:**

利用组织芯片技术和免疫组织化学SP法检测54例正常肺组织、287例NSCLC原发癌组织以及103例淋巴结转移癌组织中CRABPII和E-FABP的表达。

**结果:**

CRABPII在NSCLC原发癌组织中的表达与患者的性别、肿瘤的有无转移、TNM分期有关（*P* < 0.05）。E-FABP在NSCLC原发癌组织中的阳性表达率分别高于正常肺组织和淋巴结转移癌组织（*P* < 0.05）。在NSCLC原发癌组织中，E-FABP的表达与肿瘤的病理分级、有无转移有关（*P* < 0.05）。在NSCLC中，E-FABP的阳性表达较CRABPII占优势（*P* < 0.05），两种蛋白的差异性表达与肿瘤的大小、病理分级、有无转移、TNM分期有关（*P* < 0.05），瘤体愈大，肿瘤发生转移，临床分期愈晚，E-FABP的表达愈占优势。*Kaplan-Meier*单因素生存分析显示：CRABPII的表达、CRABPII与E-FABP的差异性表达与NSCLC患者的预后有关（*P* < 0.05）。

**结论:**

E-FABP在NSCLC中高表达，其表达的增强可能与NSCLC的发生和演进有关；CRABPII可能在NSCLC的发展过程中起负向调节作用，CRABPII阴性表达患者术后生存率更高，对NSCLC患者预后的评估有一定价值。

## Abstract (en)

**Background and objective:**

The cellular retinoic acid-binding protein Ⅱ (CRABPⅡ) and epidermal fatty acid-binding protein (E-FABP) both serving as the transport protein of retinoic acid (RA), through RA signal transduction pathway, commit the cell to opposite fate, apoptosis or survival. The aim of this study is to investigate the expression of CRABPII and E-FABP and significance in non-small cell lung cancer (NSCLC) and their lymph node metastases with tissue microarray technique.

**Methods:**

CRABPII and E-FABP proteins were detected in 54 normal lung tissues, 287 primary NSCLC tissues and 112 lymph node metastatic tissues by SP method of immunohistochemical staining.

**Results:**

The expression level of CRABPII in the primary lesions was relevant to the gender of NSCLC patients, the metastasis and TNM staging of NSCLC (*P* < 0.05), while the expression level of E-FABP was related to the grading and metastasis of NSCLC (*P* < 0.05). The positive expression rate of E-FABP in NSCLC primary lesion was remarkably higher than that in adjacent normal lung tissues and lymph node metastases, respectively (*P* < 0.05). The expression level of E-FABP had a recognizable advantage over that of CRABPII in NSCLC primary lesions (*P* < 0.05). The differential expressions between CRABPII and E-FABP was correlated with the tumor size, grading, metastasis, TNM staging of NSCLC (*P* < 0.05). The larger the tumor was and the later the TNM staging was, along with cancer metastasis, the more likely the expression of E-FABP would dominate. The expression of CRABPII and difference expression between CRABPII and E-FABP were closely related to the prognosis of NSCLC patients based on *Kaplan-Meier* survival analysis.

**Conclusion:**

E-FABP showed high exp ression in NSCLC, and the increased E-FABP expression may involved in the occurrence and development of NSCLC. CRABPII might have a negative effect in NSCLC progression, and its expression was negatively related to the prognosis of NSCLC patients.

肺癌对人类健康的威胁日益严重，目前肺癌患者的5年生存率仅为15.6%^[1]^，深入研究肺癌的分子生物学特征对肺癌的早期诊断、治疗及改善预后具有重要意义。最新研究^[2]^显示，细胞视黄酸结合蛋白（cellular retinoic acid-binding protein Ⅱ, CRABPⅡ）和表皮型脂肪酸结合蛋白（epidermal fatty acid-binding protein, E-FABP）作为维甲酸（retinoic acid, RA）的转运蛋白，将RA从细胞质转运至细胞核，分别与两种不同的核受体－－维甲酸受体（retinoic acid receptor, RAR）和过氧化物酶体增殖物激活受体β/δ（peroxisome proliferator-activated receptor β/δ, PPARβ/δ）相互作用，在RA信号通路上发挥不同的作用，从正反两方面影响细胞的增殖和凋亡。本实验利用组织芯片技术和免疫组织化学染色检测CRABPII和E-FABP在非小细胞肺癌（non-small cell lung cancer, NSCLC）原发癌组织及其淋巴结转移癌组织中的表达情况，探讨两者在NSCLC中表达的意义及其与NSCLC发生发展的关系。

## 1 材料与方法

### 1.1 材料

收集四川大学华西医院1998年1月-2003年12月手术切除、临床资料完整和尚有石蜡标本的原发性NSCLC 287例，其中伴有转移143例，实际收集到其淋巴结转移灶103例。另取54例正常肺组织作为对照。287例NSCLC标本均经切片、HE染色，明确病理诊断（2003年WHO肺肿瘤组织学分类标准）。其中，男性230例，女性57例，男:女为4.04:1。发病年龄35岁-82岁，中位年龄61岁。鳞状细胞癌133例，腺癌126例，腺鳞癌28例; 高分化癌13例，中分化癌144例，低分化癌102例，未包括腺鳞癌。根据TNM分期，Ⅰ期93例，Ⅱ期61例，Ⅲ期108例，Ⅳ期25例（2009年肺癌国际分期修订版）。患者截止术前均未行放疗或化疗。随访时间0.06个月-97+个月，平均随访时间37个月，中位随访时间35个月。生存期的计算从手术日期起到随访日期或由于复发、转移而死亡的日期为止。

### 1.2 方法

#### 1.2.1 组织芯片的制作

选择所需病例存档蜡块，根据HE染色切片进行形态学观察，确定具有代表性的病变部位（避开出血、坏死、明显炎细胞浸润及纤维化区域）后在组织切片和相应石蜡组织块上标志。将市售石蜡溶化后，注入模具内，制取大小为40 mm×30 mm×10 mm的载体蜡块。在载体石蜡上用组织芯片制备仪的打孔针打出间距为1.5 mm，直径为1.0 mm-1.2 mm，深度为4 mm的孔，设计成9×7阵列。用采样针从已标志好的组织中获取直径为1.0 mm-1.2 mm的组织块并将其压入已打好孔的载体石蜡中。NSCLC原发癌、淋巴结转移癌与正常肺组织随机排列，左上角留一空白孔作为定位标识。每例肿瘤组织取3个-5个点，每例正常肺组织取3个点。

#### 1.2.2 免疫组织化学染色试剂及方法

羊抗人CRABPII多克隆抗体和大鼠抗人E-FABP单克隆抗体分别购自美国Stata Cruze和R & D公司。免疫组化SP试剂盒和DAB显色试剂盒均购自北京中杉金桥生物技术有限公司。CRABPII和E-FABP一抗的工作稀释浓度分别为1:400和1:200。本实验采用SP法作免疫组织化学染色，具体步骤严格按照产品说明书进行。分别用正常乳腺组织和银屑病皮肤组织作为CRABPII和E-FABP的阳性对照，以0.01 mol/L PBS（pH7.4）代替一抗作为阴性对照。

#### 1.2.3 免疫组织化学染色结果判定

CRABPII和E-FABP均以细胞质或/和细胞核呈黄色颗粒为阳性。高倍镜下随机选取10个视野共记录1, 000个阳性细胞，综合染色强度和阳性细胞数量进行判定。①按切片中细胞着色深浅评分：0分为细胞无显色; 1分为黄色; 2分为棕黄色; 3分为棕褐色。②按阳性细胞率评分：0分为 < 5%;1分为5%- < 25%;2分为25%- < 50%;3分为50%- < 75%;4分为≥75%。取①、②两项评分的乘积作为总积分：0分为阴性（-）; 1分-4分为弱阳性（+）; 5分-8分为中等强度阳性（++）; ≥9分为强阳性（+++）。

#### 1.2.4 统计学方法

采用SPSS 16.00软件包进行统计学分析。分类资料采用χ^2^检验; 采用*Spearman*等级相关分析进行相关性分析; 应用*Kaplan-Meier*法进行单因素生存分析，差异显著性检验采用对数秩检验（*Log-rank test*）; *Cox*比例风险回归模型进行多因素生存分析。*P* < 0.05为差异有统计学意义。

## 2 结果

### 2.1 CRABPII和E-FABP在正常肺组织、NSCLC原发癌组织及其淋巴结转移癌组织中的表达

在正常肺组织中，CRABPII均呈阴性表达（图 1）; E-FABP的阳性表达见于Ⅱ型肺泡上皮细胞和部分支气管腺体（图 2）。CRABPII和E-FABP在NSCLC原发癌组织中表达程度各异（图 1、图 2）。

**1:**
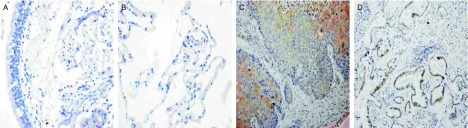
CRABPII在正常支气管粘膜上皮（A）、肺泡上皮（B）及鳞状细胞癌（C）、腺癌（D）中的表达（A, B: SP, ×400; C, D: SP, ×200） Expressions of CRABPII in normal bronchial epithelium (A), alveolar epithelium (B), squamous cell carcinoma (C) and adenocarcinoma (D) (A, B: SP, ×400; C, D: SP, ×200)

**2:**
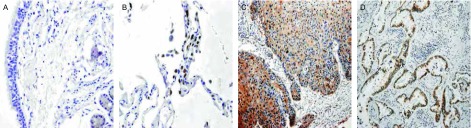
E-FABP在正常支气管粘膜上皮（A）、肺泡上皮（B）及鳞状细胞癌（C）、腺癌（D）中的表达（A, B: SP, ×400; C, D: SP, ×200） Expression of E-FABP in normal bronchial epithelium (A), alveolar epithelium (B), squamous cell carcinoma (C) and adenocarcinoma (D) (A, B: SP, ×400; C, D: SP, ×200)

CRABPII在NSCLC原发癌组织和淋巴结转移癌组织中的阳性表达率分别为39.0%和33.0%，其差异无统计学意义（χ^2^=1.171, *P*=0.279 > 0.05）。E-FABP在正常肺组织、NSCLC原发癌组织和淋巴结转移癌组织中的阳性表达率分别为24.1%、58.2%和19.4%，E-FABP在NSCLC原发癌组织中的阳性表达率分别高于正常肺组织（χ^2^=21.223, *P* < 0.001）和淋巴结转移癌组织（χ^2^=45.651, *P* < 0.001）（表 1）。

**1:** CRABPII和E-FABP在正常肺组织、非小细胞肺癌原发癌组织及淋巴结转移癌组织中的表达 Expression of CRABPII and E-FABP in normal lung tissues, non-small cell lung cancer (NSCLC) primary lesions and metastasis lymph node

Tissue type	*n*	CRABPII			E-FABP	
		(–)	(+)-(+++)		(–)	(+)-(+++)
Normal lung tissues	54	54	0		41	13
Primary NSCLC tissues	287	175	112		120	167
Metastasis lymph node	103	69	34		83	20

### 2.2 CRABPII和E-FABP在NSCLC原发癌组织中的表达及其与临床病理特征的关系

在NSCLC原发癌组织中，CRABPII的表达与患者的性别、肿瘤的有无转移以及TNM分期有关（*P* < 0.05）。CRABPII在女性患者中的阳性表达率高于男性患者; 在伴有癌转移的NSCLC中的阳性表达率低于不伴有转移的NSCLC; TNM分期越晚，CRABPII的阳性表达率越低。E-FABP的表达与肿瘤的病理分级和有无转移有关（*P* < 0.05）。E-FABP在不同病理分级NSCLC中的阳性表达率不全相同（χ^2^=15.014, *P*=0.001 < 0.05);进一步两两比较发现，E-FABP在中分化癌中的阳性表达率高于高、低分化癌（χ^2^=14.565, *P* < 0.001）; 在高分化癌与低分化癌之间的表达差异无统计学意义（χ^2^=0.441, *P*=0.507 > 0.05）。E-FABP在伴有癌转移的NSCLC中的阳性表达率高于不伴有癌转移的NSCLC（表 2）。

**2:** CRABPII和E-FABP在NSCLC原发癌中的表达与临床病理特征的关系 Relationship between expression of CRABPII and E-FABP and clinicopathological characteristics of NSCLC

Characteristic	*n*	CRABPII		*P*	E-FABP		*P*
		(–)	(+)-(+++)		(–)	(+)-(+++)	
Gender				0.019			0.726
Male	230	148	82		95	135	
Female	57	27	30		25	32	
Age (year)				0.320			0.416
≤61	154	98	56		61	93	
> 61	133	77	56		59	74	
Tumor size				0.336			0.835
Diameter ≤3 cm	108	62	46		46	62	
Diameter > 3 cm	179	113	66		74	105	
Histological type				0.157			0.809
Squamous cell carcinoma	133	89	44		58	75	
Adenocarcinoma	126	70	56		50	76	
Adenosquamous carcinoma	28	16	12		12	16	
Grading				0.060			0.001
Well	13	4	9		6	7	
Moderate	144	89	55		45	99	
Poor	102	66	36		57	45	
Metastasis				0.004			0.019
Yes	143	99	44		50	93	
No	144	76	68		70	74	
TNM staging				0.004			0.531
Ⅰ+Ⅱ	154	82	72		67	87	
Ⅲ+Ⅳ	133	93	40		53	80	

### 2.3 CRABPII和E-FABP在NSCLC原发癌组织中表达的关系

#### 2.3.1 CRABPII和E-FABP在NSCLC原发癌组织中的表达比较

在NSCLC原发癌中，CRABPII和E-FABP的表达在（-）、（+）、（++）、（+++）中的分布不同，E-FABP的阳性表达较CRABPII占优势，其差异有统计学意义（χ^2^=40.090, *P* < 0.001）（表 3）。

**3:** CRABPII和E-FABP在NSCLC原发癌组织中的表达关系 Relationship between expression of CRABPII and E-FABP in primary NSCLC lesions

Group		E-FABP				Total
		–	+	++	+++	
CRABPII	–	61	86	18	10	175
	+	47	10	19	10	86
	++	12	9	3	2	26
	+++	0	0	0	0	0
Total		120	105	40	22	287

#### 2.3.2 CRABPII和E-FABP在NSCLC原发癌组织中的差异性表达与各临床病理学特征的关系

在NSCLC原发癌组织中，219例E-FABP的表达强于或相当于CRABPII（E-FABP≥CRABPII），即E-FABP和CRABPII的表达强度呈–/–、+/–、+/+、++/–、++/+、++/++、+++/–、+++/+、+++/++、+++/+++; 68例E-FABP的表达弱于CRABPII（E-FABP < CRABPII），即E-FABP和CRABPII的表达强度呈-/+、+/++、-/++、++/+++、+/+++、-/+++。CRABPII和E-FABP在NSCLC原发癌组织中的差异性表达与肿瘤的大小、有无转移、TNM分期和病理分级有关（*P* < 0.05）。NSCLC肿瘤愈大、伴有转移、TNM分期愈晚，E-FABP的表达愈占优势。E-FABP表达强于或相当于CRABPII的在不同病理分级NSCLC中的构成比不全相同（χ^2^=6.655，*P*=0.036 < 0.05）; 进一步两两比较发现，高分化癌中E-FABP的表达强于或相当于CRABPII的构成比低于中、低分化癌（χ^2^=4.251, *P*=0.039 < 0.05），中分化癌和低分化癌中其构成比的差异无统计学意义（χ^2^=2.501, *P*=0.114 > 0.05）（表 4）。

**4:** CRABPII和E-FABP在NSCLC原发癌中的差异性表达与各临床病理特征的关系 Relationship between difference expression of CRABPII and E-FABP and clinicopathological characteristics of NSCLC

Characteristic	*n*	Difference expression between CRABPII and E-FABP		*P*
		E-FABP≥CRABPII	E-FABP < CRABPII	
Gender				0.118
Male	230	180	50	
Female	57	39	18	
Age (year)				0.332
≤61	154	121	33	
> 61	133	98	35	
Tumor size				0.034
Diameter ≤3 cm	108	75	33	
Diameter > 3 cm	179	144	35	
Histological type				0.163
Squamous cell carcinoma	133	108	25	
Adenocarcinoma	126	92	34	
Adenosquamous carcinoma	28	19	9	
Grading				0.036
Well	13	7	6	
Moderate	144	118	26	
Poor	102	75	27	
Metastasis				0.029
Yes	143	117	26	
No	144	102	42	
TNM staging				0.018
Ⅰ+Ⅱ	154	109	45	
Ⅲ+Ⅳ	133	110	23	

### 2.4 CRABPII与E-FABP在NSCLC原发癌组织中表达的相关性

*Spearman*非参数等级相关分析显示，CRABPII和E-FABP在NSCLC原发癌组织中的表达无相关性（*r*=-0.051, *P*=0.386 > 0.05）。

### 2.5 CRABPII与E-FABP的表达与预后的关系

#### 2.5.1 *Kaplan-Meier*单因素生存分析

将CRABPII和E-FABP的表达分为阴性和阳性两组; CRABPII与E-FABP的表达差异分为E-FABP的表达强于或相当于CRABPII组（E-FABP≥CRABPII）和E-FABP的表达弱于CRABPII组（E-FABP < CRABPII）。采用*Kaplan-Meier*法绘制NSCLC患者生存曲线，*Log-rank*检验不同样本的生存曲线，结果显示：CRABPII阳性表达组NSCLC患者的生存率高于阴性表达组（χ^2^=6.443, *P*=0.011 < 0.05）（图 3）; E-FABP不同表达水平NSCLC患者的生存率差异无统计学意义（χ^2^=1.664, *P*=0.197 > 0.05）（图 4）; E-FABP表达强于或相当于CRABPII组的生存率低于E-FABP表达弱于CRABPII组的患者（χ^2^=8.632, *P*=0.003 < 0.05）（图 5）。

**3:**
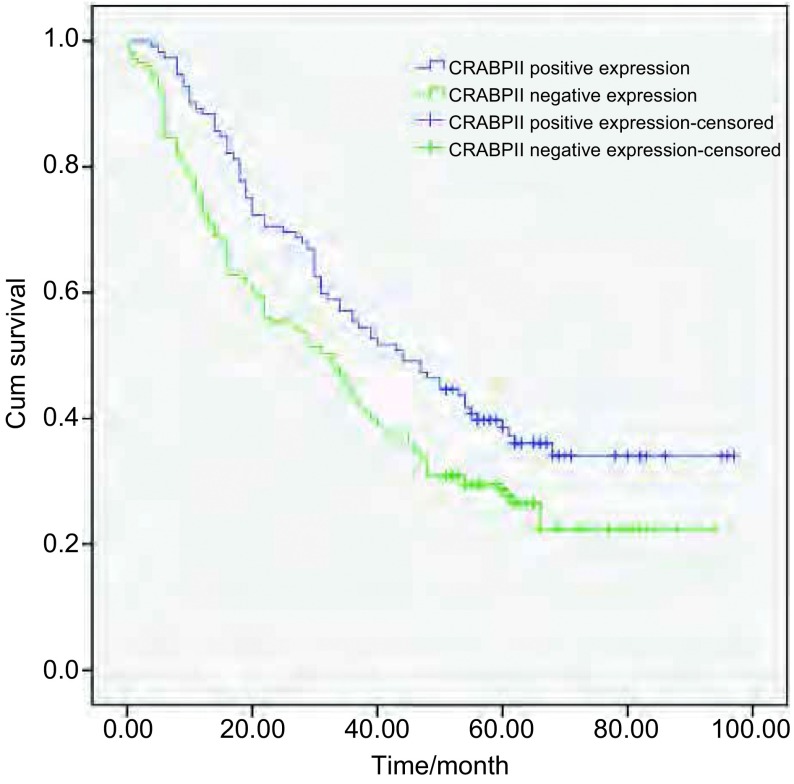
CRABPII阴性表达组和阳性表达组NSCLC患者的生存曲线 The survival curves of NSCLC patients with negative and positive expression of CRABPII

**4:**
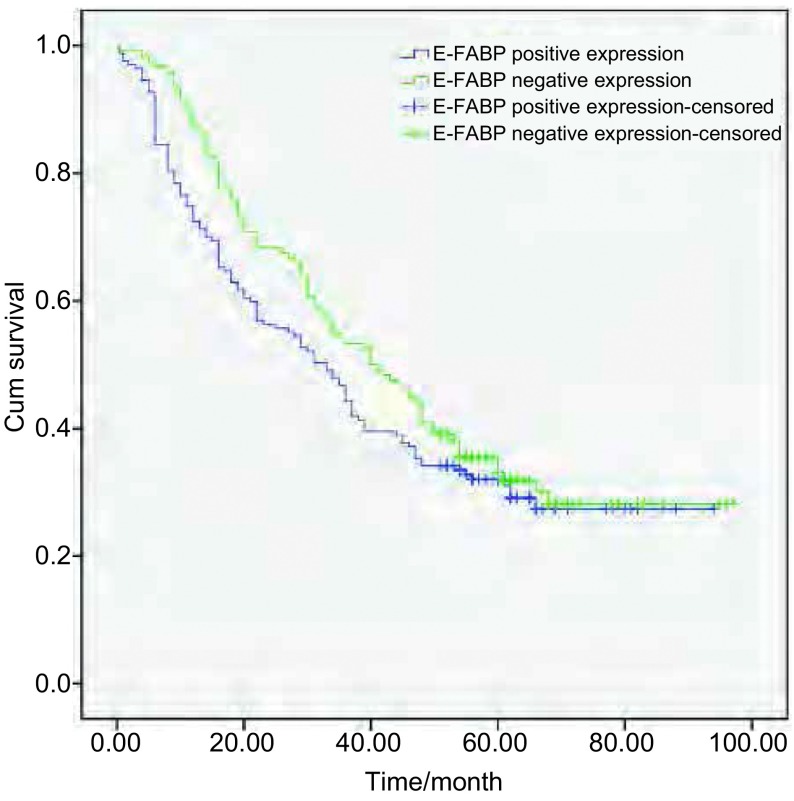
E-FABP阴性表达组和阳性表达组NSCLC患者的生存曲线 The survival curves of NSCLC patients with negative and positive expression of E-FABP

**5:**
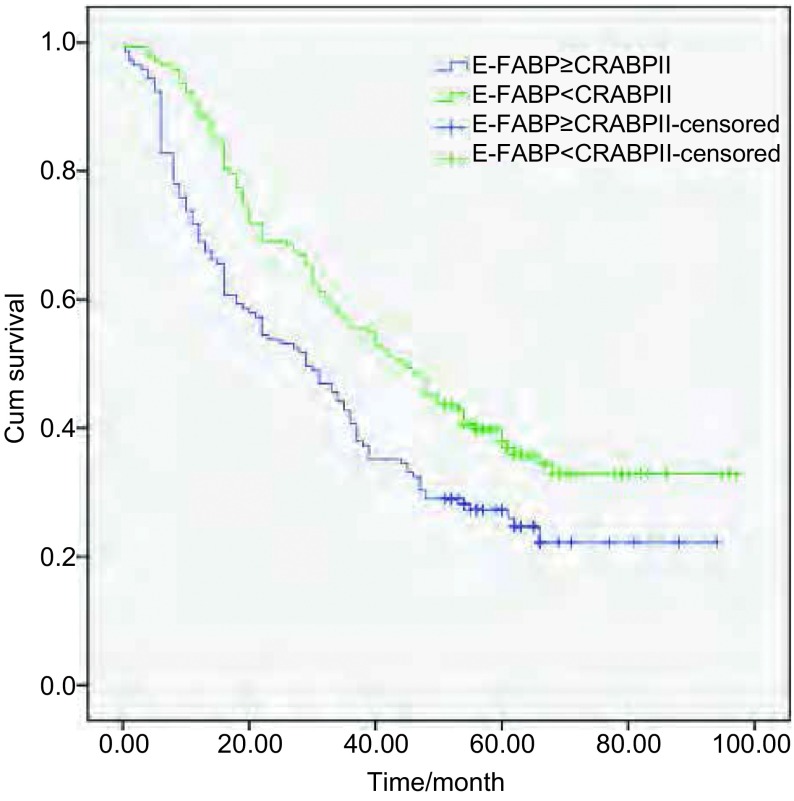
CRABPII与E-FABP差异性表达的NSCLC患者生存曲线 The survival curves of NSCLC patients with difference expression between CRABPII and E-FABP

#### 2.5.2 *Cox*比例风险回归模型分析

通过*Cox*比例风险回归模型进行多因素预后分析，包括NSCLC患者的年龄、性别、肿瘤的大小、组织学类型、病理分级、TNM分期、有无癌转移、CRABPII和E-FABP的表达以及CRABPII与E-FABP的差异性表达。采用前向逐步法，在α=0.05的水平上，肿瘤的大小（*P*=0.024）、有无癌转移（*P*=0.040）和TNM分期（*P*=0.001）这3个因素被筛入*Cox*模型内（表 5），是影响NSCLC患者预后的独立危险因素。NSCLC的肿瘤直径 > 3 cm的患者其死亡风险是直径≤3 cm患者的1.448倍; 伴有癌转移的患者其死亡风险较不伴有癌转移患者增加了64%;随着TNM分期的递增，患者的死亡风险依次增加53.4%。

**5:** *Cox*回归模型筛选的影响NSCLC患者的危险因素 The risk factors of NSCLC patients selected by *Cox* regression model

Risk factor	β	SE	Wald	*P*	Exp(β)
Tumor size	0.370	0.164	5.127	0.024^*^	1.448
Metastasis	0.495	0.241	4.227	0.040^*^	1.640
TNM staging	0.428	0.132	10.452	0.001^*^	1.534

## 3 讨论

细胞视黄酸结合蛋白（cellular retinoic acid-binding proteins, CRABPs）是调节RA和RAR之间相互作用的重要物质，包括CRABPI和CRABPII。CRABPI通过影响与RA代谢有关的酶从而调节RA的代谢，CRABPII与RA的转录活性有关^[3]^。CRABPII作为RA的转运蛋白，其主要作用是结合RA，并将RA转运至细胞核，与RAR相互作用，调节转录，从而发挥RA调节细胞分化^[4]^、调控细胞周期^[5]^、诱导细胞凋亡^[6]^，进而抑制细胞生长的作用。*CRABPII*基因在肿瘤中和正常组织中的表达存在差异，其在精囊腺中的高表达被认为可能是精囊腺不易发生肿瘤的原因^[7]^。在乳腺癌小鼠动物模型中，CRABPII能抑制肿瘤细胞的生长和转移^[8]^。本研究发现在NSCLC原发癌中，CRABPII的表达与患者的性别、肿瘤有无转移和TNM分期有关。CRABPII在女性患者中的阳性表达率高于男性患者，这可能与雌激素有关，雌激素能诱导并调节CRABPII的表达^[9]^。CRABPII的表达还与肿瘤细胞的侵袭能力有关，CRABPII在伴有转移的NSCLC中的阳性表达率低于无转移的NSCLC; 而且CRABPII的表达随TNM分期的上升而下降的趋势，并且与NSCLC患者的预后呈负相关。本实验结果提示，在NSCLC中，CRABPII可能具有抑制肿瘤细胞的转移和肿瘤演进的作用，尚有待进一步深入研究证实。

E-FABP与脂肪酸的摄取、转运和代谢有关，并且作为信号分子参与调节细胞的分化和基因的表达^[10]^。近年来多项研究发现，很多在肿瘤细胞和正常细胞中差异性表达的基因，其表达的增加或缺失与肿瘤的发生和演进有关，而*E-FABP*就是这样一种基因^[11, 12]^。在肺鳞状细胞癌^[13]^、肝细胞癌^[14]^、前列腺癌^[15]^中，E-FABP均较相应正常组织高表达。另外，*E-FABP*基因是一个肿瘤转移诱导基因，能够诱导肿瘤细胞发生转移^[16]^。E-FABP不仅与肿瘤的发生有关，而且还参与肿瘤的演进。本实验结果显示，在正常肺组织中，E-FABP的阳性表达见于Ⅱ型肺泡上皮细胞和部分支气管腺体。E-FABP与表面活性物质的合成有关，具有维持表面活性物质平衡的作用^[17]^。在NSCLC原发癌组织中，E-FABP的表达与肿瘤的有无转移和病理分级有关。E-FABP在NSCLC组织中的阳性表达率高于正常肺组织，提示E-FABP可能参与了NSCLC的发生。E-FABP在有转移的NSCLC中阳性表达率高于无转移的NSCLC，其在NSCLC原发癌组织中的阳性表达率高于淋巴结转移癌组织。Uma等^[18]^在舌鳞状细胞癌中也发现，E-FABP mRNA在原发癌组织中的表达是其淋巴结转移癌组织中的4倍，提示在淋巴结转移癌组织中肿瘤细胞在一定程度上丢失了表达E-FABP的能力。E-FABP能促进肿瘤细胞的转移，但是在淋巴结转移癌组织中的表达却有所下降，E-FABP在转移过程中的具体调节机制有待更深入的研究。以上结果提示：E-FABP不仅参与了NSCLC的发生，还可能与NSCLC肿瘤细胞的侵袭能力有关，即能促进NSCLC肿瘤细胞的转移。

CRABPII和E-FABP作为RA的转运蛋白，调节RA与两种不同的核受体-RAR和PPARβ/δ相互作用。CRABPII将RA转运至RAR，抑制细胞生长; 而E-FABP将RA转运至PPARβ/δ，促进细胞增殖。在不同的细胞中两种结合蛋白表达水平的不同，也会引起受RA调节的相关基因表达的不同，进而引起对RA不同的效应^[2]^。CRABPII/E-FABP呈高比例时，主要通过RAR通路，发挥促凋亡作用; 相反，当CRABPII/E-FABP呈低比例时，则主要通过PPARβ/δ通路，发挥促增殖的作用。两种受体，RAR和PPARβ/δ可以存在于同一种细胞或者同一细胞中，细胞中CRABPII和E-FABP的比例关系决定RA激活何种核受体，影响细胞的增殖和凋亡。

本实验研究发现，在NSCLC原发癌组织中，CRABPII和E-FABP的表达在（-）、（+）、（++）、（+++）中的分布不同，E-FABP在NSCLC原发癌组织中的阳性表达较CRABPII占优势。CRABPII和E-FABP的差异性表达与肿瘤的大小、病理分级、有无癌转移和TNM分期有关，并影响NSCLC患者的预后，提示在NSCLC中，E-FABP的表达愈占优势，瘤体愈大，肿瘤发生转移的几率愈大，临床分期愈晚，患者的预后愈差。

虽然RA的抑制细胞生长的作用已经明确，但其在肿瘤治疗上却往往因为明显的药物细胞毒性和肿瘤发生发展过程中形成的RA抵抗而受限^[19]^。E-FABP/PPARβ/δ通路的发现也为RA应用于肿瘤治疗提供了新的启示。在CRABPII存在的情况下，生理浓度的RA足以发挥抑制细胞增殖的作用，外源性的RA并不会增强此作用; 而缺乏CRABPII，即使RA的浓度很高，RAR也不会被活化^[20]^。那么，在生理浓度RA的作用下，细胞中CRABPII/E-FABP的比例就成了影响细胞增殖的焦点。据文献报道，13-顺式-维甲酸、全反式维甲酸能逆转或者阻止癌前病变支气管粘膜鳞状上皮化生的进展，维甲酸类化合物作为肺癌化学预防药物的潜在价值已备受关注^[21]^;而体内外实验均提示E-FABP的下调能抑制细胞的致瘤和转移能力^[15, 22]^。综上，CRABPII、E-FABP以及CRABPII/E-FABP的比例关系有望成为肺癌个性化靶向治疗研究的新方向。
